# A Systematic Review of the Screening Accuracy of the HIV Dementia Scale and International HIV Dementia Scale

**DOI:** 10.1371/journal.pone.0061826

**Published:** 2013-04-16

**Authors:** Lewis John Haddow, Sian Floyd, Andrew Copas, Richard John Cary Gilson

**Affiliations:** 1 Centre for Sexual Health and HIV Research, Research Department of Infection and Population Health, University College London, London, United Kingdom; 2 Department of Infectious Disease Epidemiology, London School of Hygiene and Tropical Medicine, London, United Kingdom; Shanghai Medical College, Fudan University, China

## Abstract

**Background:**

The HIV Dementia Scale (HDS) and International HIV Dementia Scale (IHDS) are brief tools that have been developed to screen for and aid diagnosis of HIV-associated dementia (HAD). They are increasingly being used in clinical practice for minor neurocognitive disorder (MND) as well as HAD, despite uncertainty about their accuracy.

**Methods and Findings:**

A systematic review of the accuracy of the HDS and IHDS was conducted. Studies were assessed on Standards for Reporting Diagnostic Accuracy criteria. Pooled sensitivity, specificity, likelihood ratios (LR) and diagnostic odds ratios (DOR) were calculated for each scale as a test for HAD or MND. We retrieved 15 studies of the HDS, 10 of the IHDS, and 1 of both scales. Thirteen studies of the HDS were conducted in North America, and 7 of the IHDS studies were conducted in sub-Saharan Africa. Estimates of accuracy were highly heterogeneous between studies for the HDS but less so for the IHDS. Pooled DOR for the HDS was 7.52 (95% confidence interval 3.75–15.11), sensitivity and specificity for HAD were estimated at 68.1% and 77.9%, and sensitivity and specificity for MND were estimated at 42.0% and 91.2%. Pooled DOR for the IHDS was 3.49 (2.12–5.73), sensitivity and specificity for HAD were 74.3% and 54.7%, and sensitivity and specificity for MND were 64.3% and 66.0%.

**Conclusion:**

Both scales were low in accuracy. The literature is limited by the lack of a gold standard, and variation in estimates of accuracy is likely to be due to differences in reference standard. There is a lack of studies comparing both scales, and they have been studied in different populations, but the IHDS may be less specific than the HDS. These rapid tests are not recommended for diagnostic use, and further research is required to inform their use in asymptomatic screening.

## Introduction

HIV-associated neurocognitive disorders (HAND) are defined as impairment of multiple cognitive domains in association with HIV, in the absence of other causes for the impairment [1]. HAND may affect up to half of all HIV positive (HIV+) individuals, even in regions with good access to antiretroviral therapy (ART) [2], [3]. Symptomatic HAND (HIV-associated dementia [HAD] or minor neurocognitive disorder [MND]) is recommended as a reason to initiate [4], [5] or modify [4] ART in recent European and British clinical guidelines.

The “Frascati criteria” are a research classification system that define HAD, the most severe grade of HAND, as impairment in at least two cognitive domains, scoring at least 2 standard deviations (SD) below demographically-appropriate means, with marked impairment of activities of daily living (ADL) caused by the cognitive deficits [1]. The two milder grades of HAND, much more common than HAD, are MND (defined as at least 1 SD below the mean in two domains with at least moderate impairment of ADL) and asymptomatic neurocognitive impairment (ANI) (defined similarly to MND but without impairment of ADL).

Fulfilment of the Frascati criteria requires neuropsychological (NP) testing of at least five cognitive domains from a possible seven, assessment of ADL, and exclusion of other diagnoses. The criteria are further limited by the lack of a standardised method of grading ADL, uncertainty about the clinical significance and possible oversensitivity for mild impairment [6], and lack of confirmatory neuropathological, imaging or laboratory biomarkers. The 1991 American Academy of Neurology (AAN) criteria are simpler to use, in that they only require that clinical diagnosis is “supplemented by” neuropsychological assessment, but are otherwise very similar to the Frascati criteria [7]. The 1998 Memorial Sloan-Kettering (MSK) criteria are based largely on clinical assessment and therefore may be more subjective, and are suited to an era prior to the availability of ART when HAD was terminally progressive [8].

Given the complexity of diagnosis, there is a role for rapid tests that can be incorporated into routine asymptomatic screening. The HIV Dementia Scale (HDS) was developed in 1995 as a “brief but sensitive instrument to identify [HIV-associated] dementia” [9]. The scale comprises four tests of subcortical cognitive domains (attention, motor speed, construction, and working memory). In response to culturally-specific elements of the HDS and difficulties with the administration of the anti-saccadic errors test, the International HIV Dementia Scale (IHDS) was developed as an alternative in 2005 [10]. Both tests provide a score but have a standardised cut-off for determining a positive or negative result. Both were proposed as rapid tests for screening (i.e. for individuals free of significant symptoms) and not diagnostic tests to confirm disease in patients with signs or symptoms of HAND, and patients who test positive with either the HDS or IHDS should undergo further assessment for diagnosis [9], [10]. Other brief clinical screening tools [2], [11]–[19] and computerised cognitive test batteries [20]–[22] have been used, but there are fewer studies of their accuracy.

The HDS and IHDS have been used in recent clinical studies in North and Central America [23], [24], sub-Saharan Africa [12], [25], South Asia [26], [27] and Europe [2], [28], [29], and have been considered for inclusion as screening tools in expert HIV treatment guidelines [19], [30] (although the IHDS has recently been replaced with a three-symptom questionnaire in updated European guidelines [4]), but important questions remain. First, they were devised for identifying HAD, and their performance in detecting milder neurocognitive impairment may be quite different. Second, it is unknown whether one scale has better accuracy than the other. And third, the study methods, settings and estimates vary considerably between diagnostic accuracy studies. To enable evidence-based use of these tests in clinical practice, we conducted a systematic review to estimate the accuracy of each scale for the diagnosis of HAD and MND when compared to standard diagnostic criteria.

## Methods

### Search strategy and selection criteria

A literature search was conducted in July 2011 and repeated in January 2013 by the first author, including PubMed and PsycInfo indexes, searchable online HIV/AIDS conference proceedings, specialist journals, and major online sources of HIV-related information. Search terms were formulated to capture all studies using the HDS or IHDS alongside another diagnostic method for HAND in a sample of HIV positive adults (Table 1). Manual searches included reference lists of relevant articles identified in automated searches, conference proceedings, and requests for unpublished data to authors of major articles. PubMed and PsycInfo searches were limited to 1994 onwards (the year prior to publication of the HDS) and conference abstracts were limited to available years (mainly 2001 onwards).

**Table 1:** Search terms.

“HIV” OR “HIV infections [MeSH]” OR “Acquired Immunodeficiency Syndrome [MeSH]” OR “AIDS Dementia Complex [MeSH]”
AND
“AIDS Dementia Complex [MeSH]” OR “dementia” OR “cogniti*” OR “neurocognitive” OR “neuropsych*” OR “encephalopath*” OR “encephaliti*” OR “dementia [MeSH]” OR “executive function [MeSH]” OR “cognition [MeSH]”
AND
“diagnos*” OR “sensitiv*” OR “specific*” OR “accura*” OR “scale” OR “score” OR “predictive value” OR “likelihood ratio” OR “sensitivity and specificity [MeSH]” OR “ROC curve [MeSH]” OR “predictive value of tests [MeSH]” OR “diagnosis [MeSH]” OR “neuropsychological tests [MeSH]” OR “intelligence tests [MeSH]”

These search terms were for PubMed, the primary source of citations. Searches of other data sources used modified versions of these terms.

From this initial search, studies were excluded if they duplicated data reported in another study in the search, and were only included if they used either the HDS or IHDS to assess individual HIV+ adults, as well as an appropriate reference standard for comparison. In this review, the highest-quality reference standard was a standardised clinical definition (Frascati, AAN, or MSK) supported by a NP battery evaluating at least five broad cognitive domains (attention and working memory; verbal and/or visual learning and recall; processing speed; executive functions; motor skills). Studies using other reference standards such as a detailed NP battery only, clinical opinion or brief NP tools were reviewed but not included in all stages of the analysis (see below).

### Assessment of study quality

Data collected for each study included study identifiers, the year(s) in which the work was conducted, geographical region, details of HIV positive study participants (number, age, education, degree of immunodeficiency, ART coverage, drug and alcohol use, psychiatric conditions, and relevant co-morbidities), test of interest (HDS or IHDS), reference standard for comparison, possible sources of bias and error, and the results of the test of interest and reference standard. Authors of papers with useable data were contacted to clarify their methodology.

Possible sources of bias and error were identified from a pre-specified list of quality criteria, based on Standards for Reporting Diagnostic Accuracy (STARD) guidelines [31]. Criteria to assess selection methods were the target population, inclusion and exclusion criteria, sampling methodology (consecutive, random, or opportunistic), information about eligible patients who were not recruited, and whether there was an *a priori* power calculation. Criteria relating to diagnostic methods included whether assessors completing the test and the reference standard were blinded to each other's assessment, adequacy and appropriateness of methods used for the reference standard, methods of ensuring validity and reliability of the assessments, and time lag or drop-outs between assessments. Studies were also assessed on whether the patient sample was adequately described, and whether there were any characteristics of the sample that might reduce its generalizability.

### Collection of screening or diagnostic accuracy data

The number of true and false positives (TP, FP) and number of true and false negatives (TN, FN) among HIV+ study subjects, using standard cut-offs for the test of interest (less than or equal to 10 for both scales), was determined. The reference standard was categorised as having either a severe or a moderate threshold. Severe threshold reference standards were those using Frascati or AAN criteria for HAD, or the MSK grading for AIDS Dementia Complex (grade 1 to 4). All three of these standards are similar in threshold, although the Frascati definition for HAD may represent the more severe end of the impairment spectrum [32]. Severe impairment in studies employing NP batteries was defined by similar criteria to HAD, namely impairment to ≥2 SD below normative means in at least two out of five cognitive domains. Moderate threshold reference standards were those that used MND (Frascati criteria), Minor Cognitive-Motor Disorder (MCMD; AAN criteria), or MSK grade 0.5 as a cut-off, with more severe impairment also included as a positive diagnosis. There is slightly less agreement between MND, MCMD, and MSK grade 0.5 than for more severe impairment [32], [33]. Moderate impairment in studies using NP batteries was defined by similar criteria to MND, namely impairment in at least two domains at a level of at least 1 SD below expected means.

If the necessary values could not be extracted from published papers, but it was apparent that the necessary source data might exist elsewhere (e.g. if test scores were reported as a continuous distribution), the corresponding author was contacted to request these data. If it was not possible to dichotomise both the test of interest and the reference standard, the study was excluded from the analysis.

The accuracy of the test of interest in each study was quantified by the sensitivity (true positive rate), specificity (1–false positive rate), positive likelihood ratio (LR+; equal to sensitivity ÷ [1−specificity]), negative likelihood ratio (LR−; equal to [1−sensitivity] ÷ specificity), and diagnostic odds ratio (DOR; equal to [TP×TN] ÷ [FP×FN]). Positive and negative LR can be multiplied by the assumed odds of a diagnosis being present before conducting the test (prior odds) to determine the final odds of a diagnosis being present (posterior odds). According to Jaeschke *et al*, tests with LR+ >5 or LR− <0.2 provide strong evidence for or against the diagnosis, and LR+ >10 and LR− <0.1 provide convincing diagnostic evidence in most scenarios [34]. 95% confidence intervals (CI) were calculated for each measure.

### Statistical analysis

Four groups of studies were defined, according to the test of interest (separate analyses were performed for the HDS and IHDS) and the reference standard threshold (severe or moderate). Some studies reported more than one grade of impairment and therefore contributed to more than one group. Studies were then pooled if they used comprehensive criteria (Frascati, AAN or MSK), but were discounted if they used only a NP battery or a brief tool as the reference standard. Studies were not excluded on the basis of other quality criteria. Where there were two reference standards applied to the same sample, the more comprehensive standard was retained.

For each of these four pools of studies, heterogeneity between estimates of sensitivity and specificity was assessed by chi-squared tests, ignoring studies with cell sizes of <5. Heterogeneity between estimates of LRs was assessed using the I-squared measure. Reasons for heterogeneity between studies were later assessed by meta-regression of LRs with study characteristics as the independent variable.

Pooled sensitivity and specificity were then calculated as averages, weighted by sample size, and pooled LR+ and LR− were calculated using standard meta-analysis methods for risk ratios with a random effects model. These methods have the potential to underestimate test accuracy in the presence of diagnostic threshold variation; such variation was assessed using Spearman's rank test to demonstrate correlation between sensitivity and specificity [35]. A summary DOR that is constant across diagnostic threshold removes this source of error [36].

Summary DORs were calculated using the Littenberg-Moses method [37] in which the linear relationship *D* = *a*+*bS* is examined in a regression model (where *D*  =  exp(DOR) and *S*  =  logit[TPR] + logit[FPR]), with points weighted by the square root of sample size. When calculating DORs, it was possible to combine studies with severe and moderate reference thresholds, and those with detailed NP batteries as the reference standard were re-incorporated into the analysis. A continuity correction was applied, because some studies had FN or FP equal to zero.

DORs are a single composite measure of both true- and false-positive rates, and therefore less clinically useful than other measures. To assist interpretation, predicted specificity and LRs were calculated from the average sensitivity and the summary DOR.

## Results

### Studies included in the review (Figure 1; Flowchart S1)

**Figure 1:**
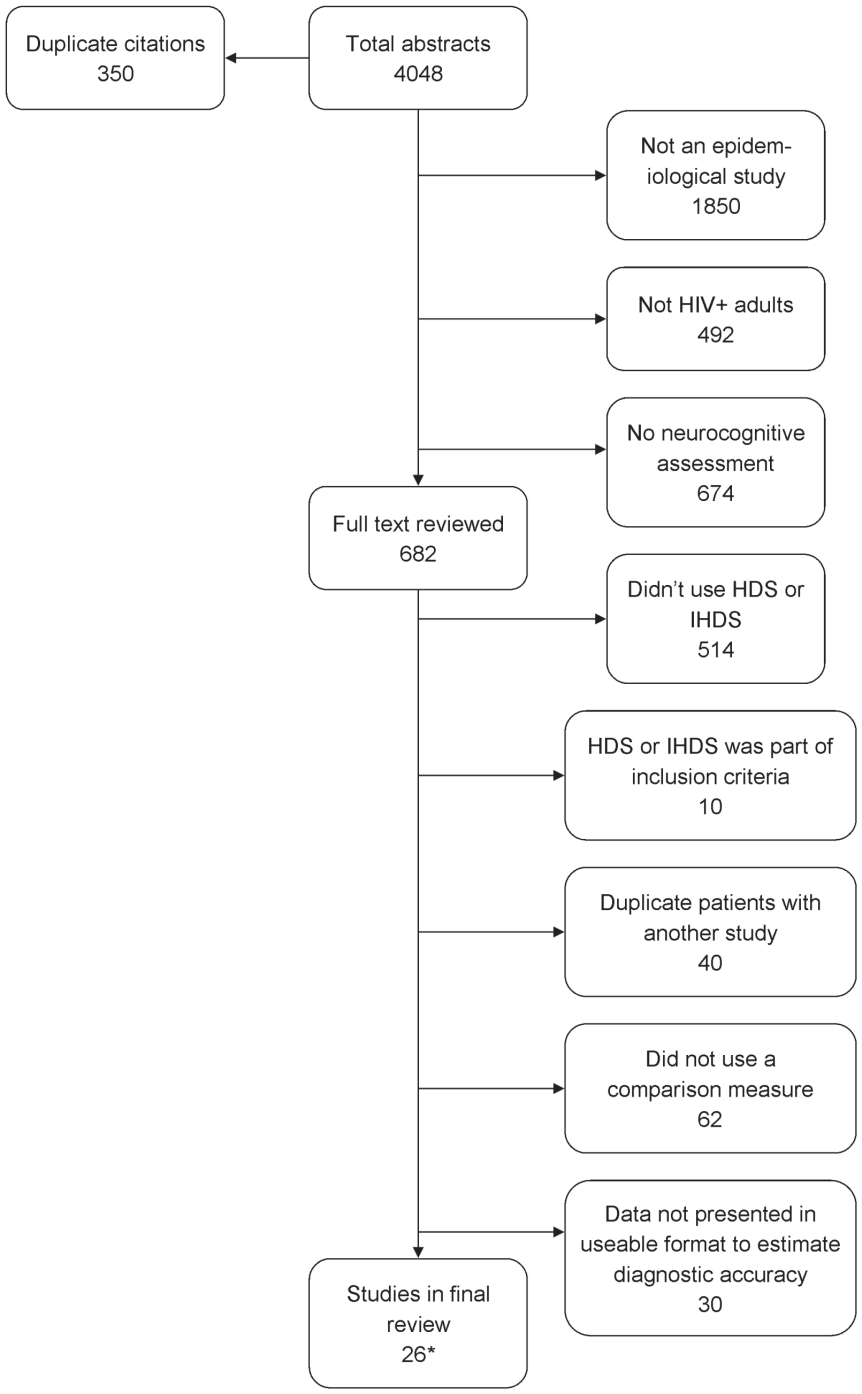
Systematic review flowchart. Footnote: * Of the final 26 studies in the review, one comprised two separate populations [10], which are treated as two different studies in all further analyses.

The literature search generated 3698 unique citations, of which 56 reported using the HDS or IHDS and a reference standard in the same HIV positive sample. Of these, 28 were discarded because they did not dichotomise both test results [17], [21], [26], [28], [38]–[61], and one was excluded because it used a non-standard cut-off [62]. A further study in rural Zambia was excluded because it was observed that all 48 HIV positive participants and all 15 HIV-negative controls scored positively (impaired) on the HDS [12]. The remainder comprised 15 studies of the HDS [2], [9], [23], [24], [63]–[73], ten of the IHDS [10], [25], [74]–[81], and one of both the HDS and IHDS [13]. One of the IHDS studies reported results from two populations and was considered as two separate studies in all further analyses [10].

Of the 27 studies meeting inclusion criteria (Table 2), several sampled high risk target populations, including patients in the pre-highly active ART (HAART) era [9], admissions to specialist AIDS facilities [64], [79], patients with low CD4^+^ counts [10], [78], [79], patients in regions with limited access to ART [10], [24], [25], [74]–[76], [78], [79], and individuals with psychiatric illness or drug abuse [71]. Nearly all studies recruited patients unselected for neurocognitive symptoms, apart from four studies that specifically targeted those with cognitive complaints [10], [76], [77] or neurology clinic referrals [13]. In contrast, two studies targeted virologically stable patients [2], [63], and two studies excluded patients known to have significant dementia [72], [75]. Eighteen studies excluded patients with confounding conditions, mainly neurological disorders (*n* = 14 studies) [2], [9], [10], [23]–[25], [63], [64], [66], [70], [76], [78], [80], psychiatric conditions (*n* = 11) [2], [10], [23]–[25], [66], [72], [76], [78], [80], systemic illnesses (*n* = 10) [2], [9], [10], [24], [63], [66], [76], [79], and drug or alcohol use (*n* = 8) [9], [23], [25], [63], [66], [72], [78]. Thirteen of the HDS studies were conducted in USA or Canada, with one HDS study in each of South Africa, Switzerland, and Puerto Rico. In contrast, only three of the IHDS studies were conducted in North America, one in India, and one in Italy, with the remainder from sub-Saharan Africa. Characteristics of the patients in HDS studies (n = 3143) and IHDS studies (n = 942) are shown in Table 3.

**Table 2:** Methods of assessment, target population, and exclusion criteria in studies of the HIV Dementia Scale (HDS) and International HIV Dementia Scale (IHDS).

Citation	Reference assessment method*	Country	Target patient population	Major exclusion criteria**
**Test of interest: HIV Dementia Scale**				
Avison [63]	MSK grading and NP battery	USA	Stable for 3 months	A, C, E
Berghuis [64]	Previous clinical diagnosis, based on AAN criteria	USA	Hospitals & community AIDS care facilities	C
Bottiggi [65]	MSK grading and NP battery	USA	On or due to receive ART	A, C, E
Carey [66]	NP battery	USA	Patients enrolled in research projects	A, C, D, E
Cloak [67]	MSK grading	USA	Unspecified HIV population	B, C, D, E
Ganasen [68]	Mini Mental State Examination	South Africa	Primary healthcare HIV clinics	NR
Gongvatana [69]	AAN criteria	USA	Unspecified HIV population	A, B, C, D
Hardy [70]	NP battery	USA	Infectious disease clinic	B, C
Morgan [23]	AAN criteria (previously applied)***	USA	Pts enrolled in research projects	A, B, C, D
Power [9]	AAN criteria (clinical assessment only)	USA	Pre-HAART era	A, C, E
Richardson [71]	NP battery	USA	Psychiatric illness or substance abuse	None
Sakamoto [73]	NP battery	USA	Broad cohort, 6 research clinic sites	None
Simioni [2]	Frascati criteria	Switzerland	Undetectable viral load, on ART	C, D
Smith [72]	NP battery	USA	Cognitively asymptomatic; unemployed	A, D
Wojna [24]	AAN criteria	Puerto Rico	Women	B, C, D
**Test of interest: International HIV Dementia Scale**				
Antinori [81]	Frascati criteria	Italy	On HAART, otherwise unspecified	NR
Joska [25]	Frascati criteria	South Africa	Primary healthcare HIV clinics	A, B, C, D
Kwasa [74]	MSK grading and Frascati criteria	Kenya	Rural Kenya	None
Meyer [75]	MSK grading and Frascati criteria	Kenya	Rural Kenya	None
Muniyandi [80]	MSK grading (clinical assessment only)	India	Medical college hospital ART clinic	C, D, E
Nakasujja [76]	MSK grading	Uganda	Off ART at baseline; pts with NCI	C, D, E
Sacktor [77]	MSK grading	USA	Progressive NCI; enrolled in a treatment trial; stable HAART	A, C, D
Sacktor [10] ****	MSK grading	Uganda	US-sponsored infectious diseases clinic	C, D, E
Sacktor [10] ****	MSK grading	USA	CD4<200, or CD4<300 with NCI	C, D, E
Singh [79]	Brief NP battery	South Africa	Ward admissions; low CD4	F
Singh [78]	Frascati criteria (using a brief NP battery)	South Africa	HIV outpatients; CD4 200–350	A, B, C, D
**Tests of interest: HDS and IHDS**				
Skinner [13]	AAN criteria (using a brief NP battery)	Canada	Neurology clinic referrals	None

* Unless stated, assessment for comprehensive clinical criteria (MSK, AAN, or Frascati) included neuropsychological evaluation of at least 5 cognitive domains.

** Major exclusion criteria were: A, past and/or recent drug and/or alcohol abuse; B, head trauma with loss of consciousness; C, neurological illness; D, psychiatric illness; E, systemic illness that may affect CNS function.

*** Participants were randomly selected from an existing cohort, in proportions approximating published prevalence of MND and HAD.

**** The paper reported two independent samples, treated as separate studies in this review.

AAN: American Academy of Neurology; ART: antiretroviral therapy; HAART: highly active antiretroviral therapy; HDS: HIV dementia scale; IHDS: international HIV dementia scale; MSK: Memorial Sloan-Kettering; NCI: neurocognitive impairment; NP: neuropsychological; NR: not reported.

**Table 3:** Characteristics of patients enrolled in studies of the HIV Dementia Scale (HDS) and International HIV Dementia Scale (IHDS).

Citation	Sample size*	CD4^+^ count, cells/μL	Mean age in years	% Male	% on ART	Reference diagnosis	% prevalence of reference diagnosis
**Test of interest: HIV Dementia Scale**							
Avison [63]	24	Median 359	Median 38 (range 29–57)	88	58	ADC grade 1 to 4	16.7
						<−2 SD on global score	20.8
						<−1 SD on global score	20.8
Berghuis [64]	103	NR	38 (range 22–62)	95	NR	HAD (AAN criteria)	11.7
Bottiggi [65]	45, 46*	NR	38.5 (SD 7.4)	87	46	ADC grade ≥0.5	52.2
						<−2 SD on global score	15.2
Carey [66]	190	47% of subjects had counts <200	41 (range 22–59)	84	NR	<−1 SD on 2/5 domains	(28.9
Cloak [67]	9	Mean 193	34 (range 16–46)	89	44	ADC grade 1 to 4	55.6
Ganasen [68]	474	Median 236	34 (range 18–62)	26	NR	≤23 points on MMSE	2.3
Gongvatana [69]	59	Mean 325 (HAD cases)	41 (SD 6.8) (HAD patients)	93	64	HAD (AAN criteria)	25.4
						MCMD	40.7
Hardy [70]	64	NR	NR	NR	NR	<−1 SD on global deficit score	34.4
Morgan [23]	135	NR	40 (SD 8)	83	63	HAD (AAN criteria)	10.4**
						MCMD	16.3**
Power [9]	137	Mean 71 in patients with MSK grade 1–2	39 (patients with HAD)	89	NR	HAD (AAN criteria)	33.6
Richardson [71]	40	Mean 502, SD 372	41 (SD 5.9)	65	NR	<−2 SD on 2/4 domains	50.0
Sakamoto [73]	1580	Median 423 (IQR 263–607)	43.1 (SD 8.5)	77	70	Clinical ratings approach [86]	51.2
Simioni [2]	100***	Mean 554 in patients with cognitive symptoms	Median 46 (range 30–70)	72	99	HAD (Frascati criteria)	2.2***
						MND	13.0***
						ANI	28.0***
Smith [72]	90	NR	41	NR	NR	<−2 SD on 2/7 tests	48.9
Wojna [24]	60	Mean 223	36 (range 22–53)	0	93	HAD (AAN criteria)	26.7
						MCMD	11.7
**Test of interest: International HIV Dementia Scale**							
Antinori [81]	101	Mean 575	NR	NR	100	HAND (any grade)	39.6
Joska [25]	96	Mean 218	30 (range 18–40)	21	NR	HAD (Frascati criteria)	36.5
						MND	31.3
Kwasa [74]	30	Mean 348	40	35	NR	ADC grade 1 to 4	16.7
						MND (Frascati criteria)	30.0
Meyer [75]	237	Median 324	36	35	NR	ADC grade 1 to 4	8.0
						HAD (Frascati criteria)	0.8
						MND	16.5
Muniyandi [80]	33	NR	Range 25–50	61	NR	ADC grade 1 to 4	9.1
Nakasujja [76]	73	Mean 130	34	27	0	ADC grade 1 to 4	1.4
Sacktor [77] (Minocycline study)	102	Mean 543	51 (SD 7)	83	100	ADC grade 1 to 4	43.1
Sacktor [10] (US-based cohort)****	81	NR	37 (SD 9.4)	NR	NR	ADC grade 1 to 4	30.9
Sacktor [10] (Ugandan cohort)****	66	Mean 186 (patients with MSK grade 0.5)	44 (SD 6.6) (patients with MSK grade 0.5)	NR	NR	ADC grade 1 to 4	37.9
Singh [79] (inpatient cohort)	20	Median 35	Median 34 (IQR 30–39)	40	NR	Severe NCI	55.0
						Moderate NCI	25.0
Singh [78] (outpatient cohort)	70	Mean 267	32 (SD 8.0)	19	NR	HAD (Frascati criteria)	14.3
**Test of interest: both HDS and IHDS**							
Skinner [13]	33	Mean 348 in cases	53 in HAND cases	92 (HAND cases)	NR	MCMD or HAD (AAN criteria)	39.4

* Sample sizes refer to the number of patients with data that were useable for meta-analysis, following discussion with study authors as necessary.

** Participants were randomly selected from an existing cohort, in proportions approximating published prevalence of MND and HAD.

*** Participants were sampled to generate two equal groups (n = 50 each): those with symptoms of cognitive impairment and those without. The quoted prevalence of MND and HAD is based on extrapolation up to a larger sample (n = 200) receiving a symptom questionnaire.

**** The paper reported two independent samples, treated as separate studies in this review.

AAN: American Academy of Neurology; ADC: AIDS dementia complex; ART, antiretroviral therapy; HAD: HIV-associated dementia; HDS: HIV dementia scale; IHDS: international HIV dementia scale; IQR: inter-quartile range; MCMD: minor cognitive/motor disorder; MMSE, mini mental state examination; MND: minor neurocognitive disorder; NCI: neurocognitive impairment; SD: standard deviation (numbers refer to number of SD relative to normative means).

### Methodology and study quality

**Figure 2:**
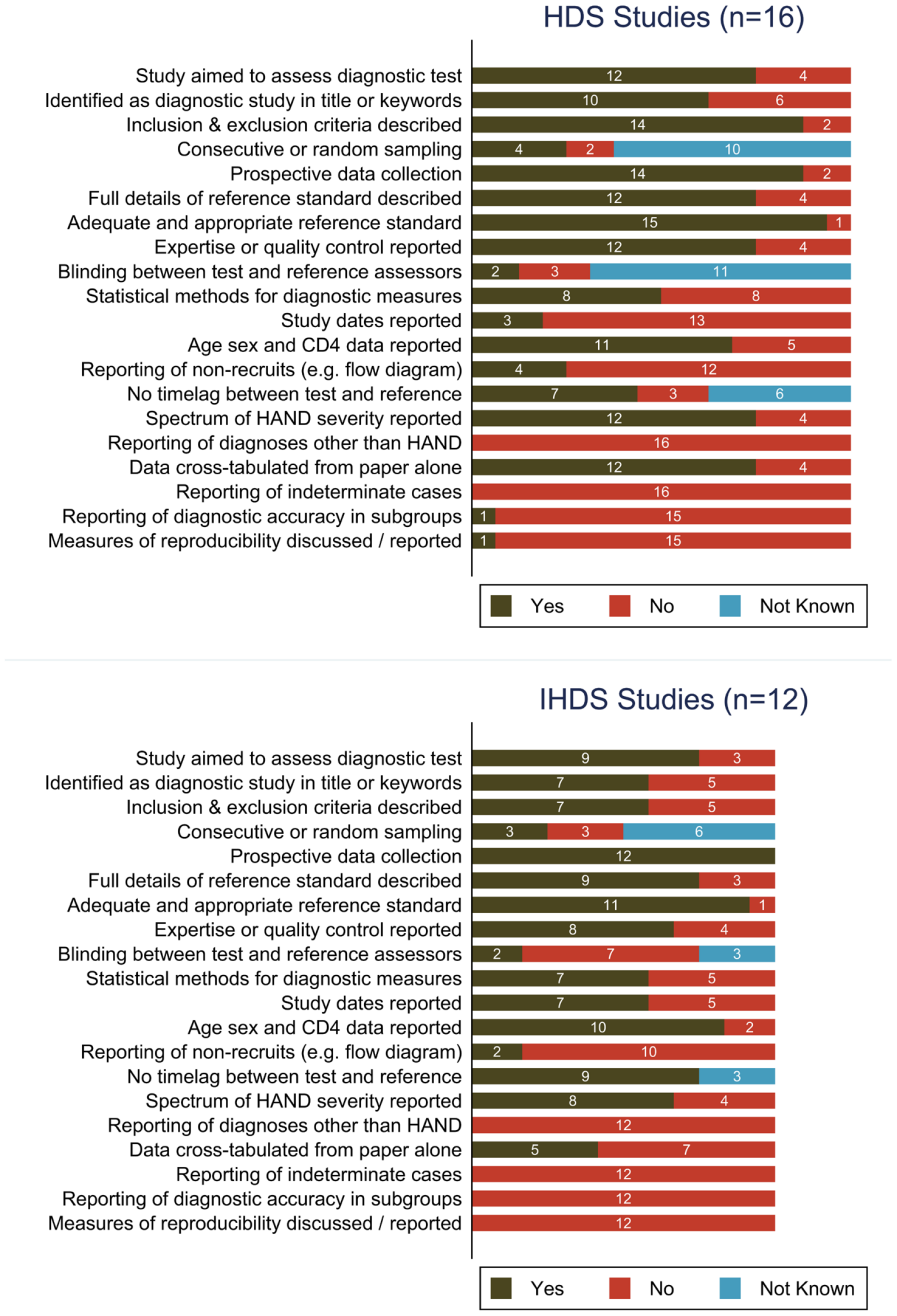
Methodology and reporting of reviewed studies: A, the HIV Dementia Scale; B, International HIV Dementia Scale. Olive-green bars indicate fulfilment of study quality criteria, red bars indicate non-fulfilment, and blue bars indicate that this feature was not reported or available from correspondence with the study author. The study by Skinner et al [13] applied both scales to the same patient sample and is represented in both graphs A and B. HAND: HIV-associated neurocognitive disorder.

Methodological characteristics relating to study quality are summarised in Figure 2. Eighteen studies were specifically designed to assess one of the screening tools [2], [9], [10], [13], [23]–[25], [64]–[66], [68], [71]–[75], [78], [79], [81]. The sampling method was random or consecutive in only seven samples (allowing for some ambiguity in reporting) [2], [23], [68], [72], [75], [79], [80]. The number of eligible patients who were not recruited was available for seven studies. No published articles reported any justification for their sample size, but one author disclosed that they had performed a power calculation [75].

Two studies used inadequate reference standards: the Mini Mental State Examination [68], or a short NP battery only [79]. Five studies used standard definitions based on clinical assessment but either did not report their NP battery [9], [64], [80] or used a battery assessing fewer than five domains [13], [79], and six studies used a NP battery only [66], [70]–[73], [81]. Norms were generally based on published demographically-standardised data, but two studies collected normative data from local HIV-negative samples [24], [25], and six studies based in Africa used norms primarily derived from US populations [10], [74]–[76], [78], [79]. Methods for quality control of the HDS or IHDS included test-retest methods [9], specialist supervision and training [25], [71], [73]–[75], [78], [79], improved standardisation [25], [71], [72], and expert panel review was used for the reference standard in some studies [10], [74]–[76].

Full [24], [69], [78], [79] or partial [23], [74], [75] blinding between assessments occurred in seven studies, and most studies did not report the use or non-use of blinding. Lack of blinding was usually due to assessments being done by the same investigator. Verification bias was difficult to exclude with available information, but three studies had a time-lag between assessments [9], [23], [64].

### Estimates of accuracy of the HIV Dementia Scale

Sensitivity estimates for detecting severe HAND with the HDS ranged widely from 35.7–91.7%, specificity 60.4–100%, LR+ 1.89–6.29, and LR− 0.12–0.72 (Table 4). After removing studies with NP batteries or brief tools as reference standards, there was evidence of heterogeneity between these estimates (p = 0.10 for LR+; p = 0.06 for LR−; p = 0.03 for sensitivity; p<0.001 for specificity). There was borderline evidence of a correlation between sensitivity and specificity across these studies (Spearman's ρ = −0.68 for nine observations, p = 0.062). Pooling seven studies that used a comprehensive reference standard gave sensitivity 68.1% (95% CI 59.2–75.9%), specificity 80.2% (76.6–83.5%), LR+ 3.76 (2.65–5.33), LR− 0.42 (0.29–0.63).

**Table 4:** Estimates of diagnostic accuracy reported in studies in the review.

Citation	Reference standard*	% Sensitivity (95% CI)	% Specificity (95% CI)	Positive likelihood ratio (95% CI)	Negative likelihood ratio (95% CI)
**Test of interest: HIV Dementia Scale, Reference standard threshold: Severe neurocognitive impairment**					
Avison [63]	MSK criteria	75.0 (30.1–95.4)	85.0 (64.0–94.8)	5.00 (1.53–16.38)	0.29 (0.05–1.62)
	NP battery	60.0 (23.1–88.2)	84.2 (62.4–94.5)	3.80 (1.08–13.41)	0.48 (0.16–1.41)
Berghuis [64]	AAN criteria, by retrospective clinical assessment	91.7 (64.6–98.5)	71.4 (61.4–79.7)	3.21 (2.22–4.63)	0.12 (0.02–0.77)
Bottiggi [65]	NP battery	71.4 (35.9–91.8)	76.9 (61.7–87.4)	3.10 (1.48–6.49)	0.37 (0.11–1.21)
Cloak [67]	MSK criteria	60.0 (23.1–88.2)	100 (50.9–100)	5.83 (0.39–88.12)	0.46 (0.17–1.25)
Ganasen [68]	Mini Mental State Examination	81.8 (52.3–94.9)	79.9 (76.0–83.3)	4.07 (2.92–5.68)	0.23 (0.07–0.80)
Gongvatana [69]	AAN criteria	53.3 (30.1–75.2)	90.9 (78.8–96.4)	5.87 (2.06–16.72)	0.51 (0.30–0.89)
Morgan [23]	AAN criteria	35.7 (16.3–61.2)	91.7 (85.5–95.4)	4.32 (1.72–10.84)	0.70 (0.47–1.04)
Power [9]	AAN criteria (clinical assessment only)	76.1 (62.1–86.1)	87.9 (79.6–93.1)	6.29 (3.53–11.21)	0.27 (0.16–0.46)
Richardson [71]	NP battery	55.0 (34.2–74.2)	75.0 (53.1–88.8)	2.20 (0.93–5.18)	0.60 (0.35–1.04)
Simioni [2]	Frascati criteria	75.0 (30.1–95.4)	60.4 (50.4–69.6)	1.90 (1.02–3.51)	0.41 (0.08–2.28)
Smith [72]	NP battery	38.6 (25.7–53.4)	84.8 (71.8–92.4)	2.54 (1.17–5.52)	0.72 (0.56–0.94)
Wojna [24]	AAN criteria	68.8 (44.4–85.8)	79.5 (65.5–88.8)	3.36 (2.65–5.33)	0.39 (0.19–0.83)
**Test of interest: HIV Dementia Scale, Reference standard threshold: Moderate-to-severe neurocognitive impairment**					
Avison [63]	NP battery	50.0 (23.7–76.3)	92.9 (68.5–98.7)	7.00 (0.96–51.09)	0.54 (0.29–1.02)
Bottiggi [65]	MSK criteria	47.8 (29.2–67.0)	90.1 (72.2–97.5)	5.26 (1.31–21.09)	0.57 (0.38–0.87)
Carey [66]	NP battery	9.1 (3.9–19.6)	97.8 (93.7–99.2)	4.09 (1.01–16.53)	0.93 (0.85–1.02)
Gongvatana [69]	AAN criteria	28.2 (16.5–43.8)	95.0 (76.4–99.1)	5.64 (0.78–40.65)	0.76 (0.61–0.94)
Hardy [70]	NP battery	40.9 (23.3–61.3)	90.5 (77.9–96.2)	4.30 (1.49–12.38)	0.65 (0.46–0.94)
Morgan [23]	AAN criteria	27.8 (15.8–44.0)	94.9 (88.7–97.8)	5.50 (2.02–15.00)	0.76 (0.62–0.94)
Sakamoto [73]	NP battery	24.4 (21.5–27.4)	91.6 (89.4–93.3)	2.89 (2.22–3.76)	0.83 (0.79–0.86)
Simioni [2]	Frascati criteria	50.0 (32.6–67.4)	62.5 (51.0–72.8)	1.33 (0.83–2.15)	0.80 (0.53–1.21)
Skinner [13]	AAN criteria (using brief NP battery)	61.5 (35.5–82.3)	80.0 (58.4–91.9)	3.08 (1.16–8.17)	0.48 (0.23–0.99)
Wojna [24]	AAN criteria	60.9 (40.8–77.8)	83.8 (68.9–92.3)	3.75 (1.68–8.37)	0.47 (0.28–0.79)
**Test of interest: HIV Dementia Scale, Reference standard threshold: Any grade of neurocognitive impairment**					
Simioni [2]	Frascati criteria**	54.1 (42.8–64.9)	96.2 (81.1–99.3)	14.05 (2.03–97.15)	0.48 (0.37–0.62)
**Test of interest: International HIV Dementia Scale, Reference standard threshold: Severe neurocognitive impairment**					
Joska [25]	Frascati criteria	57.1 (40.9–72.0)	65.6 (53.0–76.3)	1.66 (1.06–2.60)	0.65 (0.43–1.00)
Kwasa [74]	MSK criteria	100 (56.4–100)	48.0 (30.0–66.5)	1.77 (1.14–2.75)	0.17 (0.01–2.54)
Meyer [75]	MSK criteria	78.9 (56.7–91.5)	44.5 (38.1–51.1)	1.42 (1.10–1.85)	0.47 (0.20–1.14)
	Frascati criteria	100 (34.2–100)	43.0 (36.8–49.4)	1.46 (0.87–2.46)	0.39 (0.03–4.89)
Muniyandi [80]	MSK criteria (clinical assessment only)	100 (43.8–100)	40.0 (24.6–57.7)	1.67 (1.24–2.23)	0.31 (0.02–4.29)
Nakasujja [76]	MSK criteria	100 (20.6–100)	23.6 (15.3–34.6)	0.99 (0.44–2.22)	1.04 (0.09–11.91)
Sacktor [77] (Minocycline study)	MSK criteria	75.0 (60.6–85.4)	44.8 (32.7–57.5)	1.36 (1.02–1.81)	0.56 (0.31–1.00)
Sacktor [10] (US-based cohort)***	MSK criteria	80.0 (60.9–91.1)	55.4 (42.4–67.6)	1.79 (1.26–2.55)	0.36 (0.16–0.82)
Sacktor [10] (Ugandan cohort)***	MSK criteria	80.0 (60.9–91.1)	56.1 (41.0–70.1)	1.82 (1.22–2.71)	0.36 (0.16–0.82)
Singh [79] (inpatient cohort)	Brief NP battery	81.8 (52.3–94.9)	22.2 (6.3–54.7)	1.05 (0.67–1.64)	0.82 (0.14–4.71)
Singh [78] (outpatient cohort)	Frascati criteria (using brief NP battery)	70.0 (39.7–89.2)	65.0 (52.4–75.8)	2.00 (1.17–3.41)	0.46 (0.18–1.21)
**Test of interest: International HIV Dementia Scale, Reference standard threshold: Moderate-to-severe neurocognitive impairment**					
Joska [25]	Frascati criteria	53.8 (41.9–65.4)	80.6 (63.7–90.8)	2.78 (1.31–5.91)	0.57 (0.42–0.78)
Kwasa [74]	Frascati criteria	77.8 (45.3–93.7)	47.6 (28.3–67.6)	1.49 (0.87–2.54)	0.47 (0.13–1.72)
Meyer [75]	Frascati criteria	71.8 (56.2–83.5)	45.5 (38.7–52.4)	1.32 (1.04–1.66)	0.62 (0.37–1.05)
Singh [79] (inpatient cohort)	Brief NP battery	87.5 (64.0–96.5)	50.0 (15.0–85.0)	1.75 (0.65–4.74)	0.25 (0.05–1.27)
Skinner [13]	AAN criteria (using brief NP battery)	84.6 (57.8–95.7)	45.0 (25.8–65.8)	1.54 (0.97–2.44)	0.34 (0.09–1.34)
**Test of interest: HIV Dementia Scale, Reference standard threshold: Any grade of neurocognitive impairment**					
Antinori [81]	Frascati criteria**	55.0 (39.8–69.3)	82.0 (70.5–89.6)	3.05 (1.67–5.58)	0.55 (0.38–0.79)

* Unless stated, assessment for comprehensive clinical criteria (MSK, AAN, or Frascati) included neuropsychological evaluation of at least 5 cognitive domains.

** Studies using ANI as the reference standard are not included in summary estimates or figures.

*** The paper reported two independent samples, treated as separate studies.

ADC: AIDS dementia complex; ANI: asymptomatic neurocognitive impairment; CI: confidence interval; HAD: HIV-associated dementia; HAND: HIV-associated neurocognitive disorder; MCMD: minor cognitive/motor disorder; MMSE: mini mental state examination; MND: minor neurocognitive disorder; MSK: Memorial Sloan-Kettering; NCI: neurocognitive impairment; NP: neuropsychological.

Sensitivity estimates for detecting moderate-to-severe HAND were again in a wide range from 9.1–61.5%, specificity 62.5–97.8%, LR+ 1.33–7.00, LR− 0.47 to 0.93. There was also heterogeneity between estimates (p = 0.03 for sensitivity; p<0.001 for specificity; p = 0.03 for LR+), although not for LR− (p = 0.28), but little evidence of correlation between sensitivity and specificity in this pool of studies (ρ = −0.77 for six observations, p = 0.07). Pooled estimates were sensitivity 42.0% (34.6–49.7%), specificity 83.3% (78.4–87.3%), LR+ 3.18 (1.70–5.95), LR− 0.70 (0.60–0.81).

**Figure 3:**
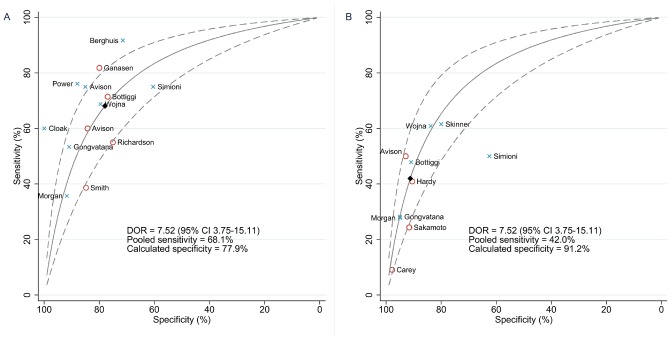
Receiver-operator characteristic curve calculated from summary diagnostic odds ratio for the HIV Dementia Scale. Blue checks indicate sensitivity and specificity estimates from individual studies using comprehensive reference standards, labelled by first author. Red circles indicate studies using neuropsychological (NP) test batteries or brief NP tests as the reference standard, again labelled by first author. Solid diamonds indicate predicted values based on pooled sensitivity and summary diagnostic odds ratio. A, Reference standard  =  AIDS dementia complex, HIV-associated dementia, or severe impairment on NP battery. B, Reference standard  =  mild neurocognitive disorder, minor cognitive/motor disorder, or moderate impairment on NP battery. CI: confidence interval; DOR: diagnostic odds ratio.

The summary DOR for the HDS was estimated at 7.52 (3.75–15.11) (Figure 3). Predictions of test accuracy for the HDS were made using the above pooled sensitivity estimates (68.1% and 42.0%), giving specificity of 77.9% for severe HAND and 91.2% for moderate-to-severe HAND, LR+ of 3.08 and 4.79, and LR− of 0.41 and 0.64, respectively. Repeat analysis using only studies with the highest-quality reference standards and populations unselected for neurocognitive symptoms gave slightly poorer accuracy estimates as follows: DOR 5.25 (1.42–19.44); sensitivity 55.6%, specificity 80.8%, LR+ 2.89, LR− 0.55 (severe HAND); sensitivity 38.0%, specificity 89.5%, LR+ 3.64, LR− 0.69 (moderate-to-severe HAND).

### Estimates of accuracy of the International HIV Dementia Scale

For the IHDS, sensitivity estimates for detecting severe HAND ranged from 57.1–100%, specificity 22.2–65.6%, LR+ 1.05–2.00, and LR− 0.31–0.82 (Table 4). Two sets of estimates came from the same study, one using MSK grading and one using Frascati criteria [75]; the latter was dropped from further analysis because the researchers found limitations to using Frascati criteria in rural Kenya. There was strong evidence of heterogeneity between studies in the specificity estimates (p<0.001), and correlation between sensitivity and specificity across IHDS studies using a valid reference standard (ρ = −0.69 for nine observations, p = 0.04), but little evidence of heterogeneity of sensitivity and LR estimates (p>0.10). Pooling studies using gave sensitivity 74.3% (67.1–80.3%), specificity 47.8% (43.9–51.8%), LR+ 1.56 (1.36–1.79), LR− 0.52 (0.40–0.68).

Sensitivity estimates for detecting moderate-to-severe HAND with the IHDS ranged from 53.8–87.5%, with specificity 45.0–80.6%, LR+ 1.32–2.78, LR− 0.25–0.62, with one conspicuous outlier of low sensitivity and high specificity [25]. There was strong evidence of heterogeneity between specificity estimates (p = 0.004), borderline evidence of heterogeneity between sensitivity estimates (p = 0.07), and no evidence of heterogeneity between LR estimates (p>0.10). There was no evidence of a correlation between sensitivity and specificity (ρ = −0.80 for four observations, p = 0.20). Pooled estimates were sensitivity 64.3% (55.6–72.1%), specificity 49.6% (43.7–55.6%), LR+ 1.73 (1.17–2.55), LR− 0.55 (0.41–0.74).

**Figure 4:**
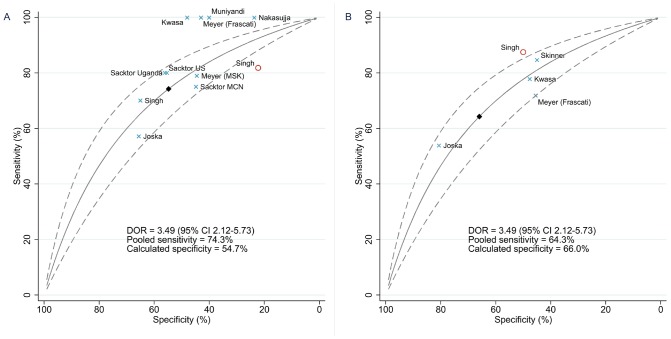
Receiver-operator characteristic curve calculated from summary diagnostic odds ratio for the International HIV Dementia Scale. Blue checks indicate sensitivity and specificity estimates from individual studies, labelled by first author. Crosses labelled “Sacktor Uganda” and “Sacktor US” correspond to two separate studies published in a single paper [10]. The cross labelled “Sacktor MCN” corresponds to baseline data from a multicentre trial of minocycline for treatment of cognitive impairment [77]. The two points labelled “Meyer” are derived from the same study [75]; “(Frascati)” and “(MSK)” denote the reference standard in each case. Red circles indicate studies using neuropsychological (NP) test batteries or brief NP tests as the reference standard, again labelled by first author. Solid diamonds indicate predicted values based on pooled sensitivity and summary diagnostic odds ratio. A, Reference standard  =  AIDS dementia complex, HIV-associated dementia, or severe neurocognitive impairment. B, Reference standard  =  mild neurocognitive disorder, minor cognitive/motor disorder, or mild/moderate neurocognitive impairment. CI: confidence interval; DOR: diagnostic odds ratio.

In summary, there was evidence of heterogeneity in specificity among IHDS studies, with considerable overlap between the ranges of estimates for detecting severe HAND and those for detecting moderate HAND. A summary ROC curve was fitted with a pooled DOR of 3.49 (2.12–5.73) (Figure 4). Predictions for the IHDS were again made using pooled sensitivity estimates (74.3% and 64.3%), giving specificity of 54.7% for severe HAND and 66.0% for moderate-to-severe HAND, LR+ of 1.64 and 1.89, and LR− of 0.47 and 0.54, respectively. Repeat analysis using only studies with high-quality reference standards and populations unselected for neurocognitive symptoms gave similar results: DOR 3.54 (2.07–6.05); sensitivity 73.4%, specificity 56.2%, LR+ 1.68, LR− 0.47 (severe HAND); sensitivity 61.9%, specificity 68.5%, LR+ 1.97, LR− 0.56 (moderate-to-severe HAND).

### Analysis of sources of heterogeneity

Analysis of study methodological features showed higher average DOR (20.5 vs. 6.85, p = 0.001) and lower average LR− (0.26 vs. 0.59, p = 0.01) in two studies comparing the HDS to a severe-impairment reference standard when the target population was highly immunodeficient [9], [64]. When compared to the IHDS, the HDS had a significantly higher summary DOR (p = 0.009) and LR+ (p = 0.019), but both scales had similar LR− (p = 0.98). This comparison may however be based on an artificial foundation, given the differences between target populations in studies of each scale. The single study that used both scales in the same population was of small sample size and failed to find a difference between the two [13].

## Discussion

We have systematically reviewed 15 studies of the HDS, ten of the IHDS, and one that included both scales. Most studies in the review apply to the original intended role of the HDS and IHDS–screening rather than diagnosis–in that participants were not selected on the basis of symptoms. Summary estimates for the HDS as a test for HAD or an equivalent diagnosis (severe HAND) were: sensitivity 68%, specificity 78%, LR+ 3.1, LR− 0.41, but its accuracy appeared to be lower when analysis was limited to studies with high-quality reference standards and unselected populations. When using the HDS as a test for MND or equivalent (all symptomatic HAND), estimates of accuracy were: sensitivity 42%, specificity 91%, LR+ 4.8, LR− 0.64. Summary estimates for the IHDS as a test for severe HAND were: sensitivity 74%, specificity 55%, LR+ 1.6, LR− 0.47. When using the IHDS as a test for all symptomatic HAND, estimates of accuracy were: sensitivity 64%, specificity 66%, LR+ 1.9, LR− 0.54. These summary estimates and most individual study estimates for both scales failed to achieve accepted levels of accuracy to provide strong evidence for a diagnosis of HAND [34], confirming their unsuitability for diagnostic purposes when used alone.

Comparing the two scales, the HDS had a higher DOR and LR+ than the IHDS, but the only direct comparison of both scales within the same sample failed to find a difference between the two, and was limited by its small sample size [13]. Furthermore, the two scales were studied in different settings, with most of the HDS studies conducted in North America, and most of the IHDS studies conducted in Africa. Unfortunately, while the IHDS was developed with resource-limited settings in mind, it is not free from culturo-linguistic effects. The four-word recall task (in both tests) must be modified for different languages [24], [52], and it was shown in an Indian population that education was associated with IHDS score, but HIV status was not [27]. The two scales were also studied in different years, and considerable changes in our understanding of HIV pathogenesis and treatment occurred in the decade between the publication of the HDS in 1995 and the IHDS in 2005.

Estimates of screening accuracy showed wide variation between studies, particularly for the HDS. We did not find strong evidence of a diagnostic threshold effect. However, tests of correlation used to demonstrate this effect are known to have low statistical power [36], and the reference diagnosis of HAD is complex and subject to variations of interpretation. It is therefore plausible that differences between reference standards contributed to the varying accuracy of these well-standardised diagnostic tools.

Regarding other sources of variability, an increased DOR and lower LR− was seen in two studies assessing the HDS in patients with more advanced immunodeficiency. Spectrum bias is a form of selection bias that may occur when the study population is sampled from a limited or specialised clinical setting and therefore has a narrow spectrum of disease. This form of bias could have increased sensitivity in samples of more severely-impaired patients, such as those conducted in Africa, in the pre-HAART era, or in hospital wards. Spectrum bias could also reduce specificity in those in whom it was difficult to exclude competing diagnoses, such as in resource-limited settings, or conversely increase specificity in samples with fewer competing diagnoses. Non-random, non-consecutive sampling strategies are known to lead to over-estimation of accuracy [82].

There were a number of methodological limitations to this review. First, the literature search and data extraction were carried out by a single author (LJH). Second, the literature search could have missed studies not cited in the target data sources, or articles in which it was not clear from the abstract that neurocognitive testing was done. To minimise this, researchers in the field were asked about the existence of unpublished datasets. Third, it was not always possible to generate two-by-two tables from available data, usually because HDS and IHDS scores were reported as continuous variables. In a few studies, the estimated values were not consistent with other information in the same article, suggesting other unknown errors in the results. This was despite requests for reconfigured data directly from researchers.

More importantly, the review is limited by the lack of a clinical gold standard for neurocognitive impairment in HIV, whether this be neurological criteria, neuroimaging findings, biomarkers in cerebrospinal fluid, or histopathology. The Frascati criteria are relatively detailed, objective, and appropriate for a research definition, so the analysis in this review provides the best available estimates of the accuracy of the HDS and IHDS when used as screening tools for MND or HAD. However, current data do not clearly inform clinicians of the natural history or appropriate treatment of these conditions, particularly milder impairment, and this limits our ability to predict the effects of screening.

British HIV Association (BHIVA) guidelines do not comment on screening for HAND [5], whereas the European AIDS Clinical Society (EACS) guidelines recommend a brief symptom questionnaire in all patients at regular intervals [2], [4] and a recent review made similar recommendations but did not support one screening test over another [83]. The general rule that one should minimise false positives if the confirmatory test is expensive or invasive favours the HDS over the IHDS, and the penalty for missing an asymptomatic case of HAND is arguably not high, so the lower-sensitivity test is acceptable. The prevalence of HAD was 2–4% of HIV positive individuals in recent surveys in the US and Switzerland [1]–[3], lower than the prevalence in most studies included in this review. At this low prior probability, one might confidently exclude the diagnosis with a negative HDS, but the posterior probability would be less than 15% after a positive HDS. In comparison, when used as a test for MND, a positive HDS result would give a posterior probability of 56% in the presence of a prior probability of 20%.

A screening test is an intervention that should be subject to interventional research as any other, and for it to be routinely used in clinical practice, the evidence base should address the next steps in the clinical pathway. For example, we need to evaluate how to investigate patients further, how to predict their outcome, and how to modify medical therapy in the light of a positive or negative screening test. On the tests themselves, studies are needed to determine their repeatability, intra-subject variation, and learning effects, and understand the causes of false positive and false negative results (not explored in the studies reviewed). Further studies of the HDS and IHDS should adhere to STARD guidelines. Specific settings of interest are the use of the HDS in an African or other resource-limited setting, or the IHDS in a North American or European setting with high ART coverage and relatively preserved immune function. There may be a role for studying the scales specifically in older adults, given the growing proportion of HIV+ individuals over the age of 50 [84] and their greater risk of HAND [85], although their ability to distinguish between HAND and non-HIV causes of NCI has not been assessed. One could also model theoretical screening programmes for neurocognitive impairment within HIV positive populations of known prevalence.

In conclusion, in current clinical practice, interpretation of the results of assessment with the HDS or IHDS requires an appreciation of their limited accuracy, the lack of generalisability of existing research, and the heterogeneity of estimates. The HDS appears to be more accurate overall and its higher specificity probably makes it the preferred test for detecting asymptomatic HAND, although the IHDS may be preferred in situations where sensitivity is most important, at the expense of loss of specificity. Having reviewed the evidence we advise against their further use as diagnostic tests for HAND in symptomatic patients, even in resource-limited settings, and believe that studies reporting their use should acknowledge their limited validity.

## Supporting Information

Flowchart S1 **Flowchart in PRISMA format.**
(DOC)

Checklist S1 **Checklist of PRISMA reporting standards.**
(DOC)
