# Supplementary material for: A Systematic Review of the Screening Accuracy of the HIV Dementia Scale and International HIV Dementia Scale
Source: PLoS One. 2013 Apr 16;8(4):e61826. doi: 10.1371/journal.pone.0061826 (PMC3628906; doi:10.1371/journal.pone.0061826)
Supplement: Flowchart S1 — Flowchart in PRISMA format. (DOC) [file pone.0061826.s001.doc]

**A Systematic Review of the Diagnostic Accuracy of the HIV Dementia Scale and International HIV Dementia Scale**


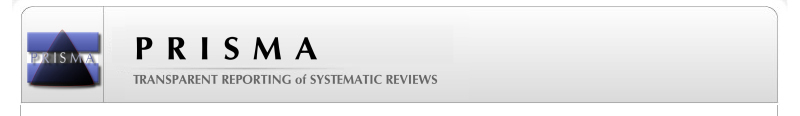
**PRISMA 2009 Flow Diagram**

**Screening**

**Included**

**Eligibility**

**Identification**

Records identified through database searching
(n = 3737 )

Additional records identified through other sources
(n = 311 )

Records after duplicates removed
(n = 3698 )

Records screened
(n = 3698 )

Records excluded
(n = 3016 )

Full-text articles assessed for eligibility
(n = 682 )

Full-text articles excluded
(n = 656 )

Studies included in qualitative synthesis
(n = 26 )

Studies included in quantitative synthesis (meta-analysis)
(n = 23 )
